# Transactions of the Medical Society of the County of Kings

**Published:** 1865-05

**Authors:** 


BUFFALO
### Medical and Surgical Journal.

VOL. IV. MAY, 1865. No. 10.

ART. I.—*Transactions of the Medical Society of the County of Kings.*

REGULAR MEETING, JANUARY, 1864.

*Fracture of the Neck of the Femur.*

Dr. Ball presented a specimen of Fracture of the Neck of the Femur. The subject was an old lady, aged 78, who, up to the time of the accident, enjoyed good health. Some time ago she fell on the hearth, causing the fracture. In course of time she was able to go about on crutches, but after death the friends feeling dissatisfied with the surgical treatment she had received, a post mortem was held, the result of which exonerated the surgeon from all censure. The fracture was found to have been *transversely* across the neck; there was some inflammatory action about the cervix, and some necrosis of the bone. The question here recurred, whether bony union will take place in fractures occurring within the capsular ligaments.

Dr. Jones related a case, where the *let alone* treatment was pursued, with as good results, as in the case in point. In forty-five days the patient was able to go about on crutches.

Dr. Willson had a similar case under observation. About five years ago the patient fell and broke one leg, and about three months ago she sustained a fracture of the other. She refused to have anything done in the way of splints, bandages, etc., but allowed *sand bags* and other appliances to be adjusted, to keep the parts quiet.

Dr. Ball desired to know the opinion of the Society, in regard to the best treatment of these fractures.

Dr. Enos remarked that no person could determine whether this fracture was *within* or *without* the capsular ligament. It was more than probable that it was partly *within* and partly *without* the ligament.

DR. SPEIR desired to know whether *exsection* of the head of the bone would give relief in any of these cases.

DR. ENOS could not recall any instance where such an operation had been performed in these fractures; he thought it would be neither proper nor judicious to undertake it, when there was neither *suppuration* nor *necrosis.**

*Diphtheria.*

DR. BELL reported two cases of scarlatina and one case of measles, which were followed by diphtheria. He regarded the debilitated state of the system as the pre-disposing cause and thought that during the prevalence of diphtheria, such patients were very liable to be attacked by it.

DR. ENOS stated in relation to these cases, that he had frequently seen cases of scarlet fever followed by measles, and vice versa; he believed there had been a simultaneous incubation of the two diseases. Most of the cases of scarlet fever he had seen lately had taken on a diphtheritic form of sore throat. He had recently seen cases of albuminuria and pneumonia, accompanied by diphtheritic deposits. Most of these cases occurred in enfeebled constitutions.

DR. REESE reported a case of mumps followed by diphtheritic deposit. The disease commenced by inflammation and swelling of the parotid gland and diphtheritic deposit in the throat; in about thirty hours inflammation of the brain set in, the swelling in the neck subsiding, convulsions then occurred and patient died. About that time another child in the same family was attacked with mumps and diphtheritic sore throat, and was treated with iron and quinia, and local treatment; this patient lived three or four days, and died of exhaustion. Dr. Bell remarked that some of the most severe cases of diphtheria he had seen were accompanied by swelling of the parotid gland; one case which he had seen with Dr. Crane died in forty-eight hours; he was convinced of the tendency of diphtheria to take hold upon debilitated constitutions or to supervene on other diseases.

DR. DODGE reported a case in a patient 56 years of age; pulse very feeble; could not be counted; patient was roused with difficulty; the respiration indicated that the membrane extended down the larynx. The next day membrane appeared upon the tonsils

* Dr. Enos is referred to report of a case of removal of the head of the thigh-bone after fracture, published in Buffalo Medical and Surgical Journal vol. iii, page 299.—ED.

and velum. Brandy, beef tea and tinct. ferri muriatis were added, and the patient gradually improved. A peculiar point in regard to this lady, was that at the age of 17, she menstruated regularly three times, but never since then. She has never suffered any inconvenience from it.

Drs. Hart, Conkling, Ball and Burge had all seen a succession of cases, not generally of grave character. Dr. Burge reported the case of a boy, six years old, in which dyspnœa was extreme for one week. He recovered gradually under the use of milk-punch, beef essence, chlorate of potash and citrate of iron and quinine internally, and externally a liniment of soap and tinct. of the root of aconite—one part of the latter to eight of the former.

Dr. Hart asked what was the value of chlorate of potash in these cases?

Dr. Ball had so little confidence in it that he had discontinued its use.

Drs. Conkling and Burge used it with great success, and would not be willing to relinquish it. Dr. Conkling thought all harsh and stimulating applications had done harm in the acute cases during the last year, but was confident he had seen good result from painting the tonsils with a solution of bromide of iodine, six drops to one ounce of syrupus gummi.

Dr. Burge had long since abandoned topical applications, except as gargles.

Dr. Hart was in the habit of using a solution of nitrate of silver as a gargle.

Drs. Hart and Conkling had seen quite a number of cases of scarlatina during the month.

Drs. Ball and Burge had hardly met with it at all.

*Sloughing Bubo.*

Dr. Burge had recently treated a case of sloughing bubo—of gonorrhœal origin, in which the loss of substance was so great as nearly to lay bare the femoral artery. The patient, a German, about twenty years of age, became much reduced, requiring a liberal exhibition of tonics, stimulants and opiates; of the latter one grain of gum opium every two hours for several days with marked benefit. The Doctor did not attempt a full and accurate report of the case, but desired only to present a few points of especial interest. The gangrene was arrested by the application of strong nitric acid, after there had been quite a copious venous hemorrhage. Four or five days subsequently arterial hemorrhage occurring, Drs. Gilfillan and Dodge (in Dr. Burge's absence from the city,) applied a compress, soaked with liquid persulphate of iron. This was carefully removed on the third day, since which time the immense cavity has a healthy aspect. The only local treatment at present is fine, clean oakum, applied several times a day. Patient improving rapidly.

### *Scarlet Fever with Diphtheria.*

Dr. Conkling had met with scarlet fever in an unusually severe form, many cases being complicated with diphtheria. In one case, a boy ten years of age, there were present at the first visit symptoms indicating scarlet fever, but no rash, and only slight redness of the fauces. At the second visit, twelve hours after, he was found pulseless, extremities cold, the surface of a purplish hue, and the tonsils, pharynx and roof of the mouth covered with a whitish exudation. He died four hours afterwards.

### *Necrosis.*

Dr. Minor reported several cases of necrosis operated upon at the Brooklyn City Hospital. First case, necrosis of the lower third of the femur, in female 28 years of age, disease of nine month's duration, there was considerable discharge from a sinus on the outer aspect of the limb leading to bare bone. It was considered expedient to cut down and remove the suspected sequestrum. A free incision was made along the thigh, and on exposing the bone two cloaca were found opening into the medullary cavity. A portion of the bone was removed by the aid of gouge and bone nippers, and was found to be eburnated and enlarged, but on getting into the medullary cavity no sequestrum was found. The wound is granulating and the patient doing well. The next was a case of necrosis in a robust young man. Another case was that of a boy who had suffered from inflammation, and from whom he had removed the first phalanx of one of his fingers. This patient now has disease of the lower third of the femur. A fourth case in which he had operated for necrosis of the sternum on a man about thirty years of age, he cut down upon the bone, saving the periosteum and knawed away the external table with bone nippers and removed the sequestrum. Patient is doing well.

These cases did not appear to be connected with struma, rheumatism or syphilis, and as to the primary cause of the trouble he had not formed an opinion, further than that inflammation of the periosteum was doubtless the first step in its progress. Loss of vitality in the bone and disease of the medullary membrane followed as an inevitable consequence of persistent periostitis. The most remarkable feature to him was the point of election. Why *necrosis* should so frequently occur *in the lower end of the femur*, he was much interested to know, and thought it a subject well worthy of future investigation.

*Hæmoptysis.*

Dr. Bell reported a case of hæmoptysis and syncope from mental shock. Patient 36 years of age, thin and tall; condition below par. On going to bed he coughed and spit up one-half ounce of blood and frothy mucus. This was followed by syncope. The patient was thought to be dying, and Dr. B. was sent for in haste. Brandy was administered and the patient recovered. Upon examination Dr. B. finds phthisis. He reported this case on account of the remarkable effect upon the system of this patient, from fear of his disease, being aware of a predisposition to it. He is now perfectly prostrated, not by the loss of blood, but from shock; is taking cod liver oil and whisky.

*Operation for Femoral Hernia.*

Dr. Enos reported a case upon which he operated for femoral hernia. A woman, 35 years of age, had a femoral hernia for some years; had worn a truss; about a week ago the truss came off, and she felt the hernia come down; bowels regular, and patient continued her occupation. She afterwards had sickness at stomach; saw a physician, and was sent to the Brooklyn City Hospital. Pulse 112; patient comfortable; no vomiting for twenty-four hours; abdomen soft, flabby; no pain. In right groin was a large inflamed tumor in the usual position of femoral hernia. Attempts had been made to reduce it. The case was considered a peculiar one on account of the undistended abdomen, want of pain, unobstructed bowels and regular pulse. It was deemed advisable to operate. Dr. Enos cut down upon the tumor and found it to be an omental hernia; the portion of omentum was discolored, but had no odor; the stricture was divided and the parts returned. Dr. Enos ascribed the lack of more marked symptoms to the omental character of the hernia. Patient took ether during the operation, and has since been troubled with bronchial irritation and cough.

*Ephemeral Fever.*

Dr. Minor related a case of a child, to which it was difficult to give a name. On Wednesday the child played as usual; at 5 o'clock slept, and at 6 o'clock she had high fever; pulse 140; skin hot; tongue fair. Thursday and Friday had slight remissions; no cough; brain, stomach and bowels normal, undisturbed until Friday, when there was nausea. Castor oil, warm bath and enema. Patient improving. He mentioned it as an instance of general fever without local lesion.

*Dropsy.*

Dr. Enos reported a case of dropsy. The patient is asthmatic, and has an obscure affection of the heart and lungs; no vascular lesion discovered; impeded circulation; discoloration of the lips and tongue. Dropsy commenced one month ago; extended to the abdominal cavity; limbs enormously swollen; urine free from albumen. The only source of dropsy is the obstructed circulation; patient unable to lie down, and has to bandage the forehead to procure sleep. Dr. E. punctured one of the limbs above and below the knee; after a day or two the limb not punctured burst; a swelling appeared upon dorsum of the foot; punctured it and bloody serum escaped; there is much discharge from limb; there is inflammation on both sides, but much greater on the side not punctured. He thinks this case shows the propriety of puncturing the limb before great distention occurs. The patient has been relieved by the escape of fluid, and now feels quite comfortable.

*Influenza.*

Dr. Mitchell stated that he had recently met with an unusual number of patients suffering from headache, pain in the back, restlessness, fever and coryza. He regarded the disease as influenza. After three weeks there was less of the head affection and fever, and diarrhœa occurred. In some of the cases, cold seemed to be the exciting cause, but in other cases the diarrhœa would come on in persons of good health, and who were not subject to it. He regarded the diarrhœa as the same disease.

Dr. Dodge had seen many cases of influenza lately, and noticed aphonia as a prominent symptom. He recalled three or four cases in which diarrhœa occurred. Dr. Mitchell had also noticed the occurrence of aphonia in some of his cases. Dr. Dodge finds throat affections prevalent. Dr. Ford also, but not attended with coryza.

*Pertussis.*

Dr. Mitchell related a case of whooping cough. A previous infant of the same family had died in convulsions. The present infant was attacked with whooping cough; the cough became more violent, with loss of appetite. Various remedies were used, small doses of ipecac, opiates, hydrocyanic acid and poultices to the chest. The child soon refused medicine, as it brought on coughing. They were then administered by injection; the cough increased in violence; there was but little expectoration; patient had twitchings; prognosis unfavorable, probably fatal. Dr. M. was sent for in haste, and administered ether to relieve the cough and spasm, which was very violent. This relieved the cough; there were no more spasms, and the disease was arrested. Dr. M. believes the life of the patient was saved by the use of ether. The inspirations were so few and short that it was necessary to use the ether at the commencement of a paroxysm. Dr. Mitchell was not aware when using ether in treating this case that it had been used in such cases. He now finds it has been used, but no case is recorded where the patient was under one year of age.

Dr. Ford had treated several cases of whooping cough within the past eighteen months, which commenced with unusual amount of coryza, eyes and face much swollen and acute bronchitis; the cough was unusually severe at commencement. These cases were brought to a close within three weeks by the use of mustard applications, calomel and Dover's powders, and expectorants with considerable anodynes.

Dr. Green thought that Dr. Mitchell's case confirmed the opinion that whooping cough was a spasmodic affection. He controls it by the use of belladonna extract in doses of ¼ to ½ grain in solution, every three or four hours, and sinapisms to the spine.

Dr. Dodge relies upon belladonna, but regulates the dose inversely, believing that a child can take more belladonna with impunity than an adult can. He uses extract ¼ grain, every six hours. Remembers in one instance he treated a child at the City Dispensary with belladonna and dilute nitric acid, and in four days the child was free from cough. Has repeatedly seen cases relieved by this treatment. His own child was treated in the same way, and in ten days the cough was entirely gone.

*Hematurea.*

Dr. Hallett related a case of hematurea in a boy 10 years of age. Patient was two weeks convalescent from remittent fever when Dr. H. was called to see the patient for hemorrhage from the urethra. The hemorrhage was controlled by an admintstration of persulphate of iron. There had previously been hemorrhage from the nose. The patient has pain in the urethra; passes long, thin coagula; passes his urine every ten or fifteen minutes; urine contains albumen, and under the microscope black corpuscles. Dr. H. believed the hematuria to have some connection with the remittent fever,

*Nephralgia.*

Dr. Enos related a case of nephralgia, or gravel, in a widow lady 35 years of age, of feeble constitution. She was taken several days ago with intensely severe pain in the back and right lumbar region, extending towards the anterior part of abdomen, above the crest of the ilium; this continued for several hours, subsiding sometimes, but usually only after the use of anodynes and hot fomentation. After the lapse of twenty-four hours pain recurred; forty-eight hours have now passed without any pain; blood appeared in urine, but it was difficult to say where it came from as the woman was menstruating. There was no evidence of stone. He at first supposed that the pain was produced by the passage of calculi. He remembered several cases of the same kind which recovered and no calculi were passed.

*Hysteria.*

Dr. Ford related a case of a patient who had been suffering for months from hysteria, assuming an epileptic character. Two months ago after being relieved, she was taken with severe pain in the back and shoulders; the head soon became painful; eye failed, and in three days she was perfectly blind. There was much fulness felt about the temples. After leeching the temples and blister to the spine, patient began to see again, and has been free from convulsions since. Dr. F. saw the patient yesterday, affected with the same symptoms again.

*Puerperal Convulsions.*

Dr. Conkling related two cases of convulsions in pregnant women, originating from different causes. The first patient, aged 26, six months pregnant with her second child, became during the process of gestration very plethoric, suffered with a sense of fulness and pain in the head, and was able to take but slight exercise. On a Sunday evening in April last she had a *very severe* attack of vomiting, caused by indigestible food. The stomach, after a time, became quiet, and at 1 A. M. she was quietly sleeping. The Doctor saw her again at 10 in the morning, when she was complaining of severe pain in the head. The urine was colored with blood. At 2 P. M. while alone in the room she had a convulsion which left her blind. At 4 P. M. she could not speak. The urine drawn with the catheter was thickened with blood, and hence could not be tested for albumen. Leeches were applied to the temples, and irritating injections administered. At 8 P. M. she had a severe convulsion. The Doctor then turned and delivered the child, but she continued to fail, her right side becoming entirely paralyzed, she died at 2 o'clock Tuesday morning.

The second case was that of a patient aged 40, pregnant with her sixth child. The Doctor was sent for Monday afternoon, January 7th, the messenger stating that she had had a convulsion. She was lying on the sofa, very restless, with entire loss of sight and speechless. Her feet, limbs and face were œdematous. She was put in bed, and the urine drawn with the catheter was found to be highly albuminous. She had in a short time two convulsions, which lasted longer than the previous ones. Chloroform was given, and the Doctor introduced his hand into the vagina and proceeded to dilate the os with the finger. The hand was gradually but gently crowded into the womb—the cord found to be pulsating, and the child removed alive. But a small quantity of blood was lost, the womb soon contracting firmly. Soon after a mercurial catharthic was given. Tuesday morning she remained unconscious, and the water albuminous. The cathartic acted freely during the day, and an opiate was given to quiet restlessness. Wednesday morning she could speak and see, but did not know her friends; urine slightly albuminous. Thursday morning she was entirely rational, and a mere trace of albumen in the urine. Her convalescence has been rapid and uninterrupted. In this case Dr. Andrew Otterson was present, concurring and assisting in the treatment.

In the first case there was evidently a rupture of some small blood vessels in the brain from the vomiting, and the clot thus formed occasioned the convulsions, paralysis and death. Delivery in this case was certainly uncalled for.

In the second case the indication was to remove the pressure from the venal vessels. The delivery here saved life, and undoubtedly was the only means by which that could be accomplished.

*Encysted Calculus.*

Dr. Enos reported the following case:

George Braznell, aged 30, born in England—United States 25 years, by trade a brick-layer. For seven years Braznell says he has been suffering from irritation of his bladder and urethra. The first symptoms noticed were a sudden stoppage while making a full stream—tenderness deep in the perineum and scalding sensation when making water. He sought advice at the time; had an instrument passed into his bladder, but the Doctor regarded the symptoms as the effect of a couple of gonorrhœas which he had had. Was in pretty good health at this time and for the four following years. Was treated by different practitioners, but his urinary symptoms were never removed by their treatment. Passed his usual quantity of water he thinks.

Three years since he found himself failing—getting weak and losing flesh. His urine he says contained matter—mucus, I presume—passed a good quantity. His urinary symptoms still continued the same as those above mentioned.

Two years since he was sounded by a Brooklyn Doctor, and a stone in the bladder discovered. Soon after this some other one sounded him and found a stricture. He was treated accordingly. B. says within the last year he has been failing rapidly, losing flesh and unable to do any work. His urethral symptoms continued almost the same. Passed a good deal of matter with his urine he says, and at times it [the urine] dribbled from him. The knee and ankle-joints have lately begun to swell.

November 23d, 1860, admitted into the Brooklyn City Hospital. Find, on passing a sound, a stone situated as if encysted underneath the os pubis. The patient is in a very bad condition. Pulse 100, weak. Has no appetite, very emaciated, an anxious look, knee swoollen.

December 6th, died.

From the time he came in till he died he was mostly in a drowsy state. Spent the greater part of the time asleep, and if not actually asleep, was always inclined to be so. Walking or sitting he felt drowsy. Cared for nothing except a little wine. For five or six nights prior to his death he had pretty severe sweats. His mind has been and was quite clear up to a few hours before his death.

On the morning of the 6th instant he complained of great pain in side. It was thought he had pneumonia, but a post mortem showed œdema of the lower lobe of right lung.

*Scarlatina.*

Dr. Burge stated that he had recently seen an unusually large number of cases of scarlet fever—many of them very severe. Of two deaths that occurred, one was from dropsy and the other from prostration. He was then in attendance upon a case, which in its early stages was a mild one, but in which there was effusion into the pericardium, and asked for the recommendation of appropriate treatment.

Dr. Burge also reported the case of a woman, much reduced with existing pulmonary tuberculosis and seven months pregnant, who, while attending upon her children, ill with scarlet fever, was attacked with severe sore throat, accompanied by a severe rigor, followed by a fever which soon became remittent. On the third day she miscarried, the child living eight hours. For the intense thirst he gave her a table-spoonful of liq. am. acetatis every two hours, with marked relief. She is convalescing. The Doctor thought that such a result in a case complicated with phthisis, scarlet fever, an active remittent and miscarriage, remarkable.

Dr. Ball had also seen a large number of cases of scarlet fever recently. In one case the disease itself was of a mild type. He had discontiuued his visits. After a few days he was sent for and found the patient, a girl three years of age, suffering from great irritability of the nervous system, and an entire suppression of urine. This continued, notwithstanding a varied and active course of treatment, and she died on the twelfth day of the suppression. Dr. Ball was then treating a case complicated with diphtheria.

Dr. Gardiner had seen no case of scarlet fever recently, but was attending a number of cases of diphtheria among adults. His treatment consisted of getting rid of all excrementitious matters, and then giving internally mineral acids and bitter tonics. Externally he generally applied tonics. The gargle he mostly relied upon was chlorate of potassa disssolved in one part of vinegar and two of water.

*Hemorrhage from the Bowels.*

Dr. Gardiner reported a case of passive hemorrhage from the bowels. Alteratives and Dover's powders were administered, and followed by fecal discharges and no blood. To open the bowels subsequently he gave rhubarb, which, failing, he gave Croton oil and balsam of copaiba, which brought away blood previously coagulated. During the night there were fifteen discharges. He then gave opium and gallic acid internally, and chloroform liniment was applied over the bowels externally.

Dr. Gardiner called attention to a new remedy, *Hamamelis Virginica*, or witch hazel, for the treatment of diarrhœa and neuralgia. He had used a fluid extract of it, in teaspoonful doses, with marked benefit.

*Periostitis. (?)*

Dr. Housel stated that he had been suffering, personally, for ten years with what he regarded as periostitis, resulting from the use of mercury. He is seized with intense pain in the foot or leg, or arm, at different times. The spot that is the seat of suffering, can be covered with a silver dollar. It recurred every other Friday for eighteen months. There is now an interval of a week between the attacks. No pain or soreness remains in the effected part during the interval. He had used a great variety of medicines, but had not tried quinine. He had taken such quantities of it for intermittent fever that he had an aversion to it. The iodide of potassium in thirty grain doses, every three hours, always cured it for the time.

Dr. Burge thought Dr. Housel's affection an entirely nervous one, and from its periodicity could, undoubtedly, be controlled by quinine. He had recently treated a severe case of neuralgia located in the tibia with twelve grains of quinine a day with entire success. He thought small doses of quinine preferable, as a rule, to large ones.

Dr. Ormiston remarked that he had had under treatment a case of intermittent supra orbital neuralgia, which was cured by the administration of twenty grains of quinine in divided doses, during the intermission. Smaller quantities were tried without benefit.

Dr. Burge stated that in squamous and vesicular eruptions he had used an ointment composed of ten grains of carbonate of soda to an ounce of simple ointment of nutgalls with unvarying success.

Dr. Burge inquired if, in a case of albuminuria, now under treatment, he should be justified in removing the stimulants entirely, which his patient had partaken of very freely for a long time.

In answer to this Dr. Gardiner remarked that in such a case he had stopped the use of stimulants and administered bark and the perchloride of mercury with great benefit. His patient resumed the free use of alcohol and died.

*Gun-shot Wound of the Thigh.*

Dr. Hart reported a case of gun-shot wound in the thigh. A minnie ball was supposed to have caused the wound. There were two external openings. One morning there was discharged into the poultice a lump of material looking like a firm, fibrous mass. On being opened it seemed like gunpowder wetted together. The next day another similar mass escaped from the other opening, after which the patient rapidly recovered.

*Spasm of the Leg after Labor.*

Dr. Conkling attended a lady in her ninth accouchment. Easy, natural delivery. Shortly after she was attacked with violent pain in the right leg, between the knee and ankle—leg drawn up. Laudanum injection relieved this. She had a similar attack in the two preceding labors.

*Dysmenorrhœa.*

Dr. Conkling also reported a case of dysmenorrhœa, in which there had been a membranous discharge monthly for years. Patient had been married fourteen years, and had been a great sufferer, both before and since her marriage. She had also hemorrhoids and irritation at the neck of the bladder. Profuse leucorrhœa, and at times numbness of the lower extremities. Would never until recently consent to an examination. A large, firm, smooth mass was found filling the pelvis, and apparently attached to the posterior surface of the uterus. It was painful on pressure. After gradual dilatation of the cervix, a No. 12 sound was introduced. Dr. Simpson of Edinburgh, incises the cervix instead of dilating, and then breaks up the adhesions with the finger, daily. Dr. Conkling succeeded so well with the dilatations he had no occasion to try the incision. Allusion was also made to Dr. Simpson's use of the bromide of potassium to discuss fibrous tumors in this region.

Dr. Ball suggested that this case of Dr. Conklin's might be hæmatocele.

Dr. Ball said he had lately attended in confinement a multipara with twins, still-born. The placentæ were distinct. After the delivery of the first child a placenta presented which proved to belong to the second child. Had he not supposed it to belong to the child already born he would have turned the remaining one and delivered by the feet with the hope of saving its life. The delay was considerable. Dr. Ball called this a case of *placenta previa* with the second child, in a case of twins. This gave rise to some discussion.

DR. BURGE remarked that he supposed the Doctor intended to use the expression in the popular sense, that it was of course placenta previa if the after-birth presented first, though it was quite evident it could not have been attached over the mouth of the womb, since the first child had already passed through that passage unobstructed.

*A Case of Acute Gastritis, supposed to have been caused by swallowing some acrid substance.* By DR. STEWART.

Mrs. —— was taken suddenly with an acute burning, lancinating pain in the epigastric region, attended with persistent vomiting of green matter, a small, hard, and quick pulse, a constant and urgent desire for cool drinks, and an aversion to any kind of motion; dry in the center and red on the edges. This condition was supposed to have been caused by the patient swallowing some of the juice of pickled oysters into which had been mixed some onion juice. The ejecta was always alkaline.

The patient was freely leeched over the epigastrium; a large blister applied to the same region as soon as possible. Calomel and morphia given internally. Symptoms continuing the same, gave citric acid, and followed it by Henry's magnesia, and an enema of turpentine. Soon after giving these the bowels moved, and a quantity of fecal matter came away, at which time the vomiting ceased. The pulse rallied after leeching.

On the second day the burning at the stomach continuing, gave sub. nit. bismuth and mucilaginous drinks—the latter with ice cream, was given for several days, and no other treatment required. The patient entirely recovered in about ten days.

DR. WILLSON asked why calomel was used?

DR. STEWART replied, as an antiphlogistic and to allay irritation.

*A supposed case of Severe Colic.* BY DR. HENRY.

Mr. ——, a gentleman who had been subject to colic, and had been under the Doctor's care and treatment on several occasions for the same; was taken one Sunday in church, and deeming himself competent to manage his case without a doctor's assistance, commenced operations as soon as he arrived at home; he soon became convinced that the more he did the worse the patint felt, and doubts arising as to how long this state of affairs would continue under his own management, he sent for the Doctor, who found him suffering intense pain in the right hypochondriac region. Gave him large doses of morphia, which afforded him no relief. Upon examining the abdomen found what appeared to be a cavity, but which proved to be a mere depression in the muscles, just over the crest of the illium, above which was a corresponding elevation, which proved to be a tonic contraction of the muscle, which was dissipated by firm presence.

*On the use of Chromic Acid in Internal Hemorrhoids.*
BY DR. HUTCHISON.

The disease presented the usual symptoms; the pain, however, was more severe at each menstrual period, and the patient suffered intensely at each defecation. All treatment having failed to afford her relief, and her distress being so intense, she finally consented to an operation. The Doctor removed the tumor by ligature, which was followed by relief for about one year, at which time she again suffered from the presence of more tumors. At this time nitric acid was applied thoroughly, and failed of success. Six months subsequent to this time the Doctor applied chromic acid. This application is unattended with pain, and is free from danger. Under its use, in this case, the internal tumors have disappeared, the patient entirely freed from pain, and in fact from the disease.

DR. H. inquired of the gentlemen present whether their experience in its use, in such cases, had been similar to his own?

None present had ever used chromic acid in internal hemorrhoids.

DR. JONES remarked that he had used it in—if he might be permitted to use the term—a hemorrhoidal condition of the os and neck of the uterus with good results.

*Strangulated Hernia.* BY DR. HUTCHISON.

This patient was not seen by a physician until three days after strangulation had taken place, and would not consent to be operated upon until the end of a week. The tumor was very large and somewhat discolored. Upon opening the tissues quantities of air escaped, which undoubtedly contributed largely in the make up of the size of the tumor, its presence in such amount was soon accounted for upon opening the sac and exposing the intestine, a hole of considerable size was found perforating the walls of the sphacellated portion embraced within the stricture. The stricture was released, the intestine was not returned within the cavity of the abdomen; the result was fatal.

*Operation for Hernia, and return of Sacen-bloc.*

Dr. Bell reported an operation for strangulated inguinal hernia. The tumor being as large as the two fists, and extremely tender. After dividing the stricture the sac was returned *en bloc.* The patient progressed favorably for a few days, but in consequence of bad hygiene, erysipelas set in, and on the eighth day the wound was re-opened to get rid of the *pus*, which amounted to eight ounces. By use of compressed sponge and careful bandaging, at the end of four weeks the incision had completely healed by granulations. But on the patient moving about, the adhesions broke away, and he could not assume the ubright position, without the gut coming down. The Doctor also remarked, that Dr. Detmold of New York, in an operation for femoral hernia, had intentionally effected healing by granulation for the purpose of radical cure, and thought he had succeeded. But this was for a small hernia.

*Cancer of the Lip.*

Dr. Bartlett presented a specimen and the following report of a case of cancer of the lip. The subject was a laborer, 50 years of age. When admitted into the Kings County Hospital, he was suffering from a sore on the lower lip and chin, which proved to be of a cancerous nature. The disease was of fourteen years' standing. He complained of a burning pain in the lip, especially after eating. The saliva was constantly dribbling away from the mouth, and the mucous membrane was almost wholly destroyed, as was also the integument of the chin.

After due deliberation, it was decided upon to remove the whole of the lip down to the ridge of the jaw. This was done by commencing at the angle of the mouth, on both sides, and cutting through all the tissues of the lip down to the ridge of the jaw. A transverse incision was then made along the line of the jaw, and the entire lip removed, being a portion about two inches square. Flaps were then made from the sides of the face by making an incision from the angle of the mouth, outwards and upwards, for about three inches, cutting through the platysma myoides and buccinator muscles to the masseter, which was not divided. In executing this incision, the point of the knife was kept a little higher than the handle, so as to make the mucous membrane longer than the skin, avoiding at the same time the duct of steno. The transverse incision, at the lower angle of the wound, was then prolonged outwards for about three inches, taking care to go below the facial artery as it rises over the jaw. The flaps on both sides were then dissected off, great care being necessary not to wound the facial artery, as the lip of the flap depended upon its being intact. The flaps were then brought together, and fastened by the quill suture. The incisions were all adjusted and secured with ligatures, and the mucous membrane turned over, and united to the skin, thus forming a new lip. The patient was then put to bed, and perfect rest enjoined. No untoward symptoms occurred until the third day, when secondary hemorrhage came on from the facial artery of the right side. This was controlled by pressure on the jaw. The cuts healed kindly and well; the lip was perfect, preventing entirely the dribbling of the saliva, and did not adhere to the jaw. The disease has never returned. No anæsthetics were used.

Dr. Bartlett also submitted an *enlarged aorta*, producing absorption of three dorsal vertebræ; a *fatty heart*, and *cystic disease of the kidneys.* And from another subject *two kidneys*, (one large and the other small,) showing the different stages of Bright's disease; also a *gall-bladder filled with calculi*, which produced obstruction of the duct. In this case there was no jaundice, but the urine was loaded with bile.

Dr. Heuser stated that he had, at the present time, a very interesting case of aneurism of the aorta, where the tumor was about the size of the fist. Five or six weeks before the Doctor saw him, according to the patient's statement, he complained of rheumatic pain in the axilla and shoulder, and thought it would gradually pass off. About two weeks ago the Doctor first saw him, and found the tumor as indicated.

Dr. Enos thought that cystic disease of the kidneys was owing to a change in the secreting cells of the kidneys, and not to a dilatation of the uriniferous tubules, which embrace the malpighian bodies, for the reason that the contained fluid is not urine.

### *Fracture of the Tibia.*

Dr. Hutchison related a case of oblique fracture of the tibia, occurring in a soldier, whom he had seen in Virginia. The injury was received on the 27th of June, 1862, and was treated by the surgeon in attendance. When the Doctor first saw him, on the 28th of July following, he found the bone protruding about four inches, the patient stating that the surgeon expected it would drop off. The bone was immediately removed, and on a further examination found that he had received a severe wound on the top of the head, evidently produced by buck-shot. Upon removing the detached fragments of the bones of the skull, about one-half of an ounce of pus flowed out, but whether it came from the membranes or not could not be determined. After this the Doctor lost sight of the patient until a few days ago, when he came to his office. He was then walking on a wooden leg, and the cavity in the head had filled up with bony matter.

### *Compound Dislocation of the Tibia.*

Dr. Hutchison also presented two specimens of compound dislocations of the tibia. A woman, 63 years of age, slipped on the side-walk on the 25th of February, sustaining a compound dislocation of the tibia. He saw her soon afterwards at the hospital, and removed a portion of the bone. The patient progressed favorably for a while, when erysipelas, with suppuration set in, producing complete prostration. She also suffered considerably from bed-sores. She died on the 30th of March.

The Doctor remarked, that from a careful examination of the part, he inferred that the patient would have recovered with a good limb had not erysipelas and prostration supervened.

A man, 51 years of age, of intemperate habits, fell on the side-walk, and sustained a compound dislocation of the tibia, the bone being thrown inwards. Reduced the dislocation, and prescribed opium with stimulants, to ward off, if possible, an attack of delirium tremens, to which he had been no stranger. The next day, by the advice of Drs. Minor and Buck, thought it judicious to remove the leg. At the time of the operation the patient was in pretty good condition, but on the sixth day he had retention of urine, a clammy perspiration, pinched countenance, etc. These symptoms increasing, the dressings were removed, and a very fetid discharge of pus occurred. Gangrene had already set in, and the patient died on the tenth day after the injury.

Dr. Minor remarked that he saw these cases, and was of the opinion that in all injuries of a like character, occurring in aged persons, except where the dislocation was very slight, amputation was the only thing to be thought of. In the second one of Dr. Hutchison's cases the patient was a bad subject, being intemperate in habits, and with no constitution to rely upon. He would lay it down as a rule, in all of these cases, to *amputate*, except the injury was very slight, or the patient young and robust.

Dr. Hutchison remarked, that in compound fractures, when the part is reduced, there is no violent contraction of the muscles; but in compound dislocations the reverse is the case, the muscles contracting so violently as to preclude the possibility of keeping the parts in place. In robust, strong patients, of good habits, he would prefer *resection*, as the end of the bone can be removed without producing much shock to the system. Notwithstanding the unfavorable results in the two cases related, he would still be inclined to repeat the operation of *resection.*

Dr. Enos remarked that there could be no question as to the grave nature of compound dislocation of the ankle-joint; still he thought it unwise to lay down an absolute rule, that all such cases should be amputated. We know very well that many of these limbs have been saved, an anchylosed ankle being better than an artificial limb. It is true, that in the attempt to save the foot, many lives have been sacrificed. It is the duty of the surgeon, therefore, to ascertain, if possible, what are the causes of the fatal result in conservative attempts, and where they are present, to amputate at once, remembering the instructions of Sir Astley Cooper on this subject, who had canvassed it very thoroughly, as he did every subject upon which he touched. Amputation was indicated in old persons, especially if infirm, and their recuperative energy not equal to the task of reparation. Again, if the soft parts are very much lacerated—if the blood vessels and nerves are injured very badly—if the patient, though not old, is anæmic, intemperate, or is debilitated from any cause—amputation should be performed. If, on the contrary, the patient is young or middle aged, and at the same time of good habits, vigorous and healthy, and the soft parts not badly lacerated—if sensibility remain in the foot, and the large arteries are unruptured—he thought it judicious to attempt to save the limb, either by excision or without it. If the parts cannot be readily reduced, or if reduced, and the end of the tibia slips from the astragalus, it should be taken off. If the tibia can be easily kept on the astragalus, it may be treated without *exsection.*

In both of the cases of exsection referred to, in the Brooklyn City Hospital, one under Dr. Hutchison, and the other under his own care, the patients were nearly 60 years of age. He thought Dr. Hutchison's patient would have recovered, (as you see by the specimen presented that quite an attempt at ligamentous union had occurred,) if injury of the back had not complicated the case. *Bed-sores and pneumonia* carried off the patient, after she had recovered from two attacks of phlegmonous erysipelas, attended with copious, purulent effulsion into the areolar tissue of the injured leg. This patient was, also, toothless. His own case, a man 62 years of age, was an unfortunate one. He had been temperate and in good health, with good teeth and good digestion. There was fracture of the internal malleolus, and the tibia protruded through the integument, the vessels and nerves being uninjured. Hence, notwithstanding his age, the result of the consultation was, to attempt to save the limb by removing a portion of the tibia. There was no attempt at repair; mortification set in, and the patient died in ten days. Possibly this result might have followed amputation; but this, doubtless, should have been done at once, as the vital force proved to be more feeble than had been supposed. Neither of these cases, therefore, was favorable for excision, or for conservative treatment without it.

Dr. Hutchison remarked, that in *resection* it was better to take off the whole joint, as ligamentous union would go on more rapidly.

Dr. Minor observed, that after a careful consideration of the whole subject, he could not see any hope for the patient, except in amputation.

Dr. Enos added that Sir Astley Cooper was in favor of *amputation* in compound dislocations, when the patient was very old, or where there was a great laceration of the soft parts, and injury to the nerves and vessels. His own practice would be, if the parts could be restored, and retained in their place, to let them alone; and if not, to take off the extremity of the bone. In young patients he would always resort to *exsection.*

